# *MReye-Seg*: development and validation of an automated MRI pipeline for standardised ocular and orbital morphometrics

**DOI:** 10.1038/s41433-025-04044-1

**Published:** 2025-10-14

**Authors:** Ge Tang, Seyed-Ahmad Ahmadi, Steven Jillings, Ben Jeurissen, Elena Tomilovskaya, Inna Nosikova, Alexandra Ryabova, Ekaterina Pechenkova, Viktor Petrovichev, Ilya Rukavishnikov, Theresa Velten, Marina Gripshi, Angelique Van Ombergen, Floris L. Wuyts, Peter zu Eulenburg

**Affiliations:** 1 Institute for Neuroradiology, University Hospital, LMU Munich, Munich, Germany; 2 Graduate School of Systemic Neurosciences, LMU Munich, Munich, Germany; 3 German Center for Vertigo and Balance Disorders, University Hospital, LMU Munich, Munich, Germany; 4 Lab for Equilibrium Investigations and Aerospace, University of Antwerp, Antwerp, Belgium; 5 Imec/Vision Lab, University of Antwerp, Antwerp, Belgium; 6 SSC RF – Institute of Biomedical Problems, Russian Academy of Sciences, Moscow, Russia; 7 Laboratory for Cognitive Research, HSE University, Moscow, Russia; 8 Radiology Department, Federal Center of Treatment and Rehabilitation, Moscow, Russia; 9 Department of Translational Neurosciences – ENT, University of Antwerp, Antwerp, Belgium; 10 Directorate of Human and Robotic Exploration, European Space Agency (ESA), Noordwijk, Netherlands

**Keywords:** Visual system, Visual system, Predictive markers, Prognostic markers

## Abstract

**Introduction:**

Robust and objective quantification of ophthalmic and retroorbital structures from standard neuroimaging is an accessible but still untapped resource for clinical assessment. Current approaches mostly rely on subjective manual measurements, introducing variability and limiting clinical integration. We developed and validated an automated ocular morphometrics pipeline enabling diagnostic precision through standardised orbital measurements.

**Methods:**

The presented pipeline consists of: (1) supra-resolution orbital template generation from standard structural MRI, (2) expert-guided segmentation with anatomical landmark annotation, (3) registration-based propagation to individual scans, (4) ocular neuroimaging quality control, and (5) automated extraction of 38 bilateral volumetric and geometric parameters critical for comprehensive ophthalmic and ocular structural morphometry including entire optic nerve sheath volume. We also integrated posterior globe deformation mapping for structural analysis.

**Results:**

High-resolution orbital space template generation and subsequent landmark-based segmentation succeeded for all tested T1- and T2-weighted datasets. Ocular morphometrics revealed a substantial and significant physiological asymmetry between the eyes with minimal-to-negligible inter-dataset variations for all orbital parameters and landmarks. The pipeline demonstrated excellent reproducibility with intraclass correlation coefficients exceeding 0.8 for 30/38 metrics. We then established normative ranges from adult datasets to facilitate clinical and research interpretation. Our posterior globe deformation mapping enables sensitive morphological assessment.

**Conclusions:**

This pipeline (https://github.com/Eulenburg-LMU/MReye-Seg) enables objective, standardised and comprehensive quantification of ophthalmic morphometrics, including total optic nerve sheath volume and posterior globe deformations as potential surrogates for raised intracranial pressure. By minimising measurement variability and processing time, *MReye-Seg* supports eye researchers and ophthalmologists in achieving diagnostic precision for ocular and retroorbital conditions.

## Introduction

With recent advancements in MRI technology, its application in eye and orbit imaging for scientific and clinical purposes has increased significantly. This specialised use of MRI is now referred to as *“MReye”* [1, 2].

Several new tools have been developed for segmenting the ocular and retroorbital regions from structural MR images. For instance, Ciller et al. introduced a toolbox for segmenting the sclera, the cornea, vitreous humour and lens using a statistical shape model on T1w MRIs [3]. The highly popular ITK-snap toolbox has been employed for semi-automatic segmentation of the eye and orbit on high-resolution 7 T MRIs [2].

However, most morphometric analyses still heavily rely on experienced radiologists manually adding landmarks for measurements or using conventional diagnostic criteria [4, 5], such as the Brodsky and Vaphiades imaging criteria for describing globe flattening [6]. A few studies have focused on specific aspects of the ocular structure, such as the automation of eye dimension measurements [7]. Recently, driven by interest in spaceflight-associated neuro-ocular syndrome (SANS) in space crews, Rohr et al. developed a semi-automatic processing method to measure the optic nerve (ON) and optic nerve sheath (ONS) cross-sectional area [8, 9]. However, their segmentation was based on anisotropic slice images rather than the gold-standard three-dimensional (3D) segmentation for volumetry.

To the best of our knowledge, there is currently no comprehensive processing pipeline available for the objective and fully automated analysis of ocular and retroorbital structures within the field of *MReye*. Thus, our work represents the first all-encompassing automated pipeline for targeted morphometric analysis of this anatomical region. The overarching goal and primary motivation behind the development of this sophisticated pipeline was its potential application to non-invasively assess via neuroimaging data the possibility of raised intracranial pressure after long-duration exposure to microgravity in astronauts, who have developed a syndrome known as SANS [9]. Moreover, this pipeline would have the potential to assist clinicians in the detection, diagnosis and non-invasive longitudinal monitoring of various ophthalmological and neurological disorders. Patients with idiopathic intracranial hypertension (IIH) typically exhibit an enlarged ONS [10], which may then be precisely and objectively quantified using our methodology.

Our concrete general aims were to develop an advanced automated processing pipeline designed to quantify and analyse a wide array of ocular and retroorbital morphometrics derived from suitable isotropic (3D) structural magnetic resonance imaging (MRI) data. The morphometrics as we see them should encompass four distinct categories: distance metrics, cross-sectional area metrics, geometry metrics, and volume metrics. The measurements should be based on the segmentation of key structures, including the ocular globe, lens, ON, and ONS. To validate the efficacy of our pipeline, we planned to test three different datasets from our laboratory, comprising both T1-weighted and T2-weighted MRI scans to also compare the applicability with respect to differing soft-tissue contrasts and fat-suppression techniques. And to promote open science, replicability and collaborative research, we plan to make this pipeline publicly available (https://github.com/Eulenburg-LMU/MReye-Seg) for everyone.

## Methods

**Fig. 1:**
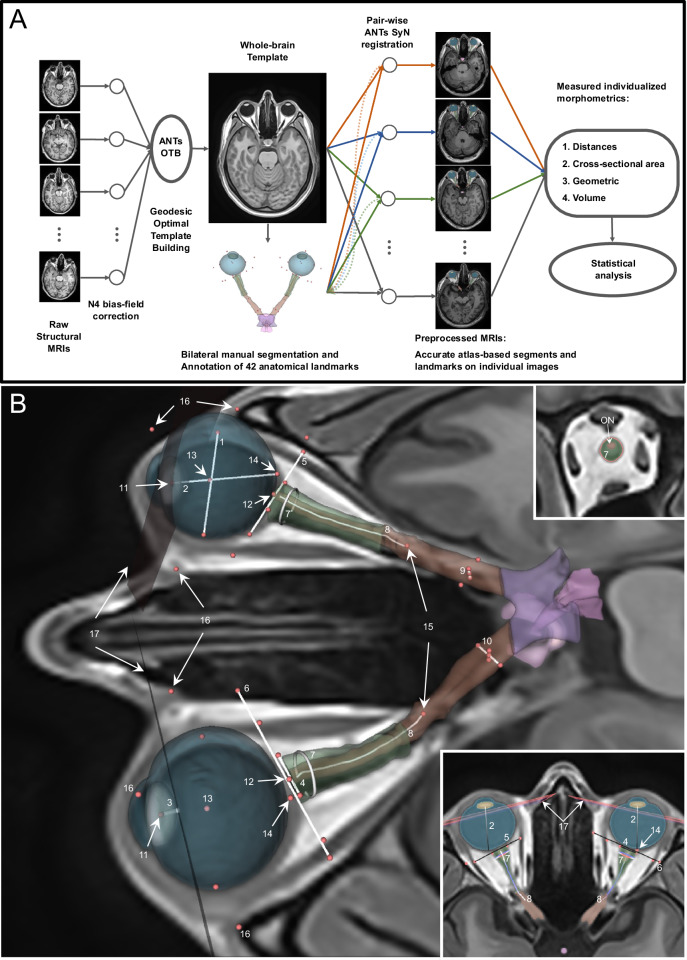
Pipeline for automated extraction of ocular and retroorbital morphometric parameters. **A** Pipeline workflow for the ocular and retroorbital morphometrics analysis. The pipeline consists of five sequential steps: (1) generation of a cohort-specific orbital template from structural MRI, (2) expert neuroradiologist-guided bilateral segmentation of ocular and retroorbital structures with precise anatomical landmark annotation, (3) registration-based propagation of segmentations from template to individual clinical MRI scans, (4) quality control for critical orbital structures, and (5) automated extraction of 38 bilateral clinically relevant quantitative morphometric parameters. **B** Segmentation and landmarks in the template. Overview of all the measured parameters for the eye, retroorbital space and optic nerve with respect to the landmarks (red dots). The right upper view represents the 3 mm-intersection plane with the optic nerve sheath, and the bottom right view depicts a transversal view where the retroorbital space landmarks for widths and planes are annotated. 1: Globe width; 2: Globe length; 3: Perpendicular point-to-plane distance of lens centre to the orbital rim (plane estimated with least-squares fit); 4: ONSH width; 5: Retroorbital space width (muscle); 6: Retroorbital space width (bone); 7: 3mm-intersection area; 8: ON centerline; 9: Optic canal minor axis; 10: Optic canal major axis; 11: Centre of the lens; 12: ON tip; 13: Centre of the ocular globe; 14: Back of the ocular globe; 15: End of the optic canal; 16: Points on bony orbital rim; 17: Estimated orbital rim plane (dark pink).

The detailed workflow of our pipeline for analysing ocular and retroorbital morphometrics is shown at Fig. 1A. The process consists of five main steps:

1. The pipeline constructs a cohort-specific template using the established ANTs geodesic optimal template building algorithm (https://github.com/ANTsX/ANTs/blob/master/Scripts/geodesicinterpolation.sh) with all structural MRIs.
2. Bilateral manual segmentation of ocular and retroorbital structures is performed on the template, followed by annotation of anatomical landmarks by an experienced neuroradiologist.
3. ANTs pair-wise SyN registration maps these segmentations and landmarks from the template to individual pre-processed MRI scans.
4. Quantitative morphometric measurements are extracted from the registered individual MRIs.
5. Statistical analyses are conducted according to specific research questions.

This systematic approach enables standardised assessment of ocular and retroorbital structures across multiple subjects while maintaining complete anatomical precision through template-based registration.

### Cohorts’ demographics and MRI protocols

We included four datasets from three different sites, consisting of 2 different sequences (T1-weighted and T2-weighted MRI).

#### Site 1 (T1-weighted)

Fifteen male participants (age range: 35–56 years, mean = 43.05 ± 6.06) were scanned longitudinally over two years at the National Medical Research Treatment and Rehabilitation Centre (Moscow, Russia), yielding 66 scans (3–6 per participant). High-resolution T1-weighted structural images (1 × 1 × 1 mm) were acquired using a GE Discovery MR750 3 T system (GE Healthcare, Milwaukee, Wisconsin) with a 16-channel receiver head coil (T1-weighted fast spoiled gradient echo; 176 slices; TR = 7.9 ms; TE = 3.06 ms; TI = 450 ms; flip angle = 12°).

#### Site 2 (T1-weighted and T2-weighted)

Twenty-six participants (18 females, 8 males; age range: 19–63 years, mean = 32.44 ± 13.07) were scanned at LMU Klinikum, Munich. High-resolution T1- and T2-weighted images were acquired using a 3 T Siemens Magnetom Prisma scanner (Siemens Healthineers, Erlangen, Germany). T1-weighted sequences used fat-suppressed water-excited fast imaging (TR = 2060 ms, TE = 2.17 ms, TI = 1040 ms, flip angle = 12°, FOV = 320 × 320 mm, voxel size = 0.75 × 0.75 × 0.75 mm, 256 slices, bandwidth = 230 Hz/Px). T2-weighted images used 3D TSE sequence (TR = 3200 ms, TE = 560 ms, FOV = 320 × 320 mm, voxel size = 0.75 × 0.75 × 0.75 mm, 256 slices, bandwidth = 230 Hz/Px).

#### Site 3 (T1-weighted)

Eight male participants (age range: 34–44 years, mean = 40.00 ± 3.35) underwent longitudinal MRI scanning over two years, yielding 63 scans (7–8 per participant). High-resolution T1-weighted 3D MPRAGE images were acquired at DLR:envihab facility (Cologne, Germany) using a Siemens mMR Biograph 3 T scanner (Siemens, Erlangen, Germany) with a 16-channel head/neck coil. Two scans were collected around one hour apart at each timepoint (TR = 1900 ms, TE = 2.43 ms, TI = 900 ms, flip angle = 9°, FOV = 256 mm, voxel size = 1 × 1 × 1 mm, 176 slices, bandwidth = 180 Hz/Px).

Ethical approval for this study was obtained from the appropriate institutional review boards at each site. Data from Site 1 were approved by the Committee of Biomedical Ethics of the Institute of Biomedical Problems of the Russian Academy of Sciences and the Human Research Multilateral Review Board (HRMRB). Data from Site 2 were approved by the medical ethics board of the LMU Hospital Munich (approval ID: 21-0705). Data from Site 3 were approved by the European Space Agency (ESA) Medical Board and the Institutional Review Board of the Antwerp University Hospital (approval number: 13/38/357). All participants provided written informed consent, and the investigations adhered to the principles outlined in the Declaration of Helsinki and its subsequent amendments (version October 2013).

### High-resolution ocular and retroorbital template building

For segmentation and landmark localisation, deformable atlas registration is currently the state-of-the-art regarding accuracy and robustness in the medical image analysis domain [11]. We built and labelled our unique dedicated templates for each dataset as we did not want to rely on one of the many publicly available and widely used T1-weighted templates, for several reasons. Firstly, we required a template that has a sharp, high-contrast representation of the regions in and around the ocular globes. This excludes the majority of templates that are typically used for neuroscientific analyses, e.g., due to skull-stripping (e.g., ATAG 7 T) [12], or because of de-facing and other anonymisation techniques (e.g., Hammers30) [13]. Some templates do contain the eye region, but in a very blurred and low-contrast representation (e.g., ICBM 2009a/b/c) [14], or they were based on one individual subject, introducing a morphological bias (e.g., Colin27-2008) [15]. Second, the source data in publicly available templates was acquired with a variety of MRI scanners than the source data in our study. This can negatively affect the accuracy and robustness of pairwise image registrations [11], since it is well known that there is a non-negligible inter-site and inter-scanner variability in MRI imaging [16, 17]. Third, the source data in our study was acquired on two scanners only with different MRI protocol (*site1_T1*, *site2_T1* and *site2_T2*). Thus, we were in the unique position to build site- and sequence-specific templates that are tailored to the respective datasets, not only in terms of scanner and head coil characteristics but also in terms of gender and age distribution.

Prior to template building, we performed a non-uniform intensity normalisation for bias field correction in all MRI scans using the N4 normalisation algorithm [18]. The template building algorithm, implemented as part of the Advanced Normalisation Tools (ANTs) toolkit [19], iteratively reconstructs an average morphology from a set of input scans. In the first iteration, all scans are rigidly aligned to an average location and rotation in a voxel grid of 1 mm isotropic resolution, and voxel intensities are computed as mean intensities. In the second and all following iterations, all input scans are registered to the current template, first in a linear-affine and then in a non-linear deformable fashion, and voxel intensities are estimated in a maximum-likelihood fashion. After a few iterations, the procedure converges to a template which is unbiased towards individual cohort members. The template represents an average cranial structure which is geodesically optimal, both regarding morphology and appearance. At its core, the template building algorithm is based on Symmetric Normalisation (SyN), a state-of-the-art registration algorithm for diffeomorphic, pair-wise alignment of MRI scans. The high accuracy and robustness of SyN registration have been validated by demonstrating state-of-the-art performance in several internationally recognised medical image processing challenges [20, 21]. To have a smooth segment for the retroorbital structure (e.g., ON, ONS), we up-sampled the 1 mm template via bspline interpolation to a 0.2 mm higher-resolution template with the Resample Scalar Volume module in 3D Slicer. To this end, in conjunction with the optimal template building implemented by ANTs, and cohort-specific templates built from matched controls, we thus realised a highly customised toolset for robust and accurate landmark localisation for individual datasets.

### Ocular and retroorbital space segmentation and landmarks annotation

The segments and landmarks were all performed within 3D Slicer 4.11 on the custom template [22]. We semi-automatically segmented the anterior visual pathways (including the ocular globe, lens, ON, ONS and optic chiasm) through intensity-based thresholding from the cohort-specific high-resolution template, with manual post-processing using the “Segment Editor” module in 3D Slicer. A consultant neurologist with extensive neuroradiological training then annotated 42 landmarks (21 landmarks per eye and retroorbital space), by careful inspection with the assistance of the 3D slicer modules (all landmarks are depicted in Fig. 1B).

Specifically, landmarks for the ON tip and retroorbital space width (at both muscle and bone levels) were positioned along tissue borders in the template volume’s axial plane. The same applied to four landmarks for measuring the optical canal area, however on an oblique plane with a perpendicular cut through the optical canal. The orbital plane was annotated with four landmarks representing the maximally inferior/superior (major axis) and medial/lateral (minor axis) extents of the bony orbital rim [23]. These points were tagged based on MRI contrasts, with supporting visual overlay from a publicly available, healthy cranial CT sample (3D Slicer toolkit) [22], which was non-linearly aligned using multi-modal ANTs SyN registration [24]. The landmark for the start of the optic canal was annotated based on the identified anatomical structure. This start point of the optic canal was also used as the end point of centerline extraction process for the ON. The centre point coordinates of the ocular globes and lenses were computed as the centre of mass of their respective segmentation maps [25]. A detailed list of anatomical landmarks and abbreviations is supplied (see Fig. 1B).

### Image registration to the template

To have the individualised morphological parameterisation, we transferred the templates, along with the segmentation maps and annotated landmarks into subject space for all acquired data via deformable image-based registration using ANTs SyN [19]. As noted, ANTs is highly robust to inter-subject differences and performs favourably in comparison to other methods [11]. To confirm successful registrations, we performed a visual inspection of the final registration results of all data for quality assessment, prior to computing distance measures.

### Quality control for the ocular and retroorbital region

In addition to the whole-brain quality control, we developed a specific quality control (QC) procedure for the ocular and retroorbital area following the registration of segmentations. This procedure employs three image quality metrics (IQMs): the Entropy Focus Criterion (EFC), Signal-to-Noise Ratio (SNR), and Contrast-to-Noise Ratio (CNR), applied to both the ocular globe and ON.

The EFC was calculated for the segmented ocular globe and ON as the regions of interest (ROI) using the following formula [26].

1 $$E=-{\sum }_{j=1}^{n}\frac{{x}_{j}}{{x}_{\max }}{\mathrm{ln}}\left[\frac{{x}_{j}}{{x}_{\max }}\right],{{\rm{with}}}\,{x}_{\max }=\sqrt{{\sum }_{j=1}^{n}{x}_{j}^{2}}$$

2 $${EFC}=\left(\frac{n}{\sqrt{n}}\log {\sqrt{n}}^{-1}\right)E$$

For SNR calculation, we utilised the ocular globe and ON segmentations as the ROI. The standard deviation of an air mask served as the background noise. The SNR was computed using the formula:

3 $${SNR}=\frac{{\mu }_{{roi}}}{{\sigma }_{{bg}}}$$

The CNR calculation followed a similar approach to the SNR, with the addition of a comparison region. This region was defined as the area surrounding the ocular globe and the ON. The CNR was calculated using the formula:

4 $${CNR}=\frac{{\mu }_{{roi}}-{\mu }_{{comparison}}}{{\sigma }_{{bg}}}$$

### Morphological parameterisation

Morphometric analysis was performed on registered individual segmentations using a custom Python script (https://github.com/Eulenburg-LMU/MReye-Seg). The analysis quantified multiple anatomical distances including lens centre to globe centre, globe centre to ON tip, globe length/width, ONS head (ONSH) width, and retroorbital space width at both muscle and bone levels. Additionally, perpendicular distances from the lens and globe centres to the orbital rim plane were computed, with the orbital rim plane fitted using least-squares estimation of four orbital rim bone landmarks.

ONS analysis was performed at a defined plane 3 mm posterior to the ON entry point at the globe, yielding two key measurements: ONS 3 mm (measured perpendicular to the ON centerline) and ONS 3 mm optimal (minimum cross-sectional area obtained through angular plane adjustments while maintaining the 3 mm distance from the entry point). These measurements were obtained using the Cross-section analysis module in 3D Slicer. The optic canal area was calculated as an ellipse based on four landmarks defining its major and minor axes. ON pathway geometry was quantified using VMTK’s Extract Centerline module [27], which generated a Voronoi diagram-based centerline with 1 mm sampling intervals, allowing computation of ON morphometric parameters including radius, length, curvature, torsion, and tortuosity.

### Deformation map of the posterior globe segment

To quantify the globe flattening, we calculated the deformation map of the ocular globe posterior segment. First, we defined a spherical coordinate system with its origin at the ocular globe centre. The zenith direction was defined as the vector extending from the ocular globe’s centre to the nerve tip, while the azimuth reference direction corresponded to the left-to-right direction of the original RAS Cartesian coordinate system used by 3D Slicer. Once the new coordinate system was defined, we transformed all points from the RAS system to the spherical system.

Afterwards, we projected every point on the segmented scleral ocular globe sphere onto the reference plane of the newly defined spherical system, which passed through the centre and was orthogonal to the zenith. Next, we converted the spherical coordinate to a 2D Cartesian system using the polar and the azimuth angles, normalising them by the maximum polar angle (90 degrees). Eventually, the radii of all points were plotted as contour levels on the deformation map of the ocular globe posterior segment.

### Statistical analysis

Statistical analyses were performed using R version 4.2.2 (R Core Team, 2022), with all analysis scripts archived for reproducibility (https://github.com/Eulenburg-LMU/MReye-Seg).

A mixed-design analysis of variance (ANOVA) was conducted using the ‘ez’ package to examine the effects of site location and asymmetry on ocular and retroorbital morphometrics [28]. The model included site as a between-subjects factor with three levels (*site1_T1*, *site2_T1* and *site2_T2*), and eye asymmetry (right versus left) as a within-subjects factor. To account for multiple comparisons, *p*-values were adjusted using the Benjamini–Hochberg procedure for false discovery rate (FDR) control. Given the exploratory nature of this study, statistical significance was set at 0.05. Post-hoc analyses were conducted for significant main effects and interactions identified in the ANOVA.

Test-retest reliability analysis was assessed using Intraclass Correlation Coefficients (ICC), calculated using the ‘irr’ package [29]. Following Bartko’s guidelines, we employed a two-way random-effects model with agreement, using single measurements as the unit of analysis [30].

## Results

### Template generation and image quality control metrics for the ocular and retroorbital space

**Fig. 2:**
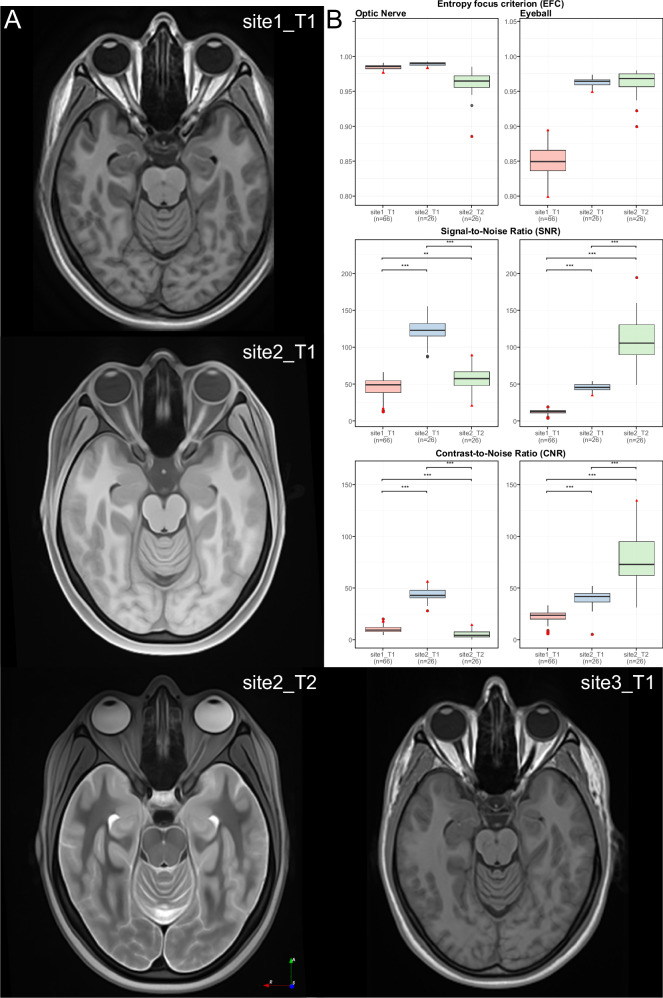
Cohort-specific templates and image quality metrics for ocular globe and optic nerve segmentation. **A** A representative axial slice from the final template for each of the four different cohorts. **B** Boxplots showing the Entropy focus criterion (EFC), Signal-to-Noise Ratio (SNR), and Contrast-to-Noise Ratio (CNR) metrics for the Optic Nerve (ON) and Ocular globe, across the dataset. The EFC demonstrates consistent movement patterns of the ON and Ocular globe within each cohort, except for six scans of ON and five scans of the Ocular globe (marked with the red triangle). The SNR and CNR demonstrated significant differences among the three cohorts, indicative of differences in the MRI protocols.

The process of template generation for the ocular and retroorbital space was successful in all datasets and cohorts (see Fig. 2a for respective template slices from the four cohorts, depicting ocular globe, ON and ONS). The *site1_T1* and *site2_T2* cohorts exhibited a clear depiction of all the relevant ocular structures. Conversely, the *site2_T1* cohort template gave a barely visible ONS, while the *site3_T1* cohort template presented with markedly diminished ON contrast. Consequently, due to the suboptimal template quality in this cohort, most likely resulting from limited sample size, the analysis was limited to regions with an excellent visibility and contrast. The remaining three cohorts were subject to the full analysis of all anatomical features.

The entropy focus criterion (EFC) was employed to quantify the distribution of principal directions, encompassing artifacts such as ghosting and blurring induced by voluntary and involuntary subject motion, for both the ON and the ocular globe. This metric facilitated the identification of motion-corrupted scans within each cohort. Our analysis revealed a limited number of statistical outliers, based on the criteria of mean ± 2 SD: three scans in the ON and four scans in the ocular globe. Following the visual inspection, we still decided to keep these outliers (The red triangle in Fig. 2b).

Statistically significant inter-group differences were observed in both signal-to-noise ratio (SNR) and contrast-to-noise ratio (CNR) metrics. The *site2_T1* cohort demonstrated better CNR in the ON region compared to the other two cohorts. on the other hand, while the *site2_T2* cohort exhibited enhanced CNR in the ocular globe relative to the remaining groups, suggesting differential tissue contrast characteristics across cohorts and anatomical structures.

### The ocular and retroorbital morphometrics

We categorised the morphometrics into four classes: distance metrics, cross-sectional area metrics, geometry metrics, and volume metrics. All reported *p*-values were adjusted using the Benjamini-Hochberg method to control the False Discovery Rate (FDR), we set a p value of 0.05 as significant.

**Fig. 3:**
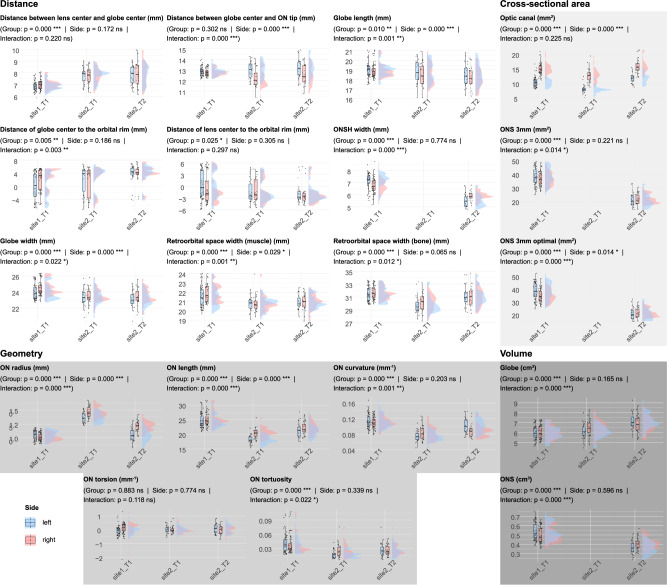
Raincloud plot visualisation of morphometric parameters with statistical analysis results. This figure presents the distribution of all measured morphometrics using raincloud plots. Each plot combines individual data points and a boxplot (left half of the plot) with a probability density function (right half of the plot) to provide a comprehensive view of the data distribution. The subtitle line displays the results of mixed two-way ANOVAs, depicting main effects for group and side, as well as their interaction effects.

Several morphometrics demonstrated significant group effects. In the distance metrics category, we observed significant differences in the distance between lens centre and globe centre (F(2,115) = 44.11, *p* < 0.001), globe length (F(2,115) = 5, *p* = 0.01), distance of globe centre to the orbital rim (F(2,115) = 6.61, *p* = 0.005), distance of lens to the orbital rim (F(2,115) = 4.25, *p* = 0.025), ONSH width (F(1,90) = 98.06, *p* < 0.001), globe width (F(2,115) = 12.19, *p* < 0.001), retroorbital space width (muscle) (F(2,115) = 15.56, *p* < 0.001), and retroorbital space width (bone) (F(2,115) = 20.48, *p* < 0.001). For cross-sectional area metrics, significant group effects were found in optic canal (F(2,115) = 27.41, *p* < 0.001), ONS 3 mm (F(1,90) = 195.43, *p* < 0.001), and ONS 3 mm optimal (F(1,90) = 177.87, *p* < 0.001). Geometry metrics showing significant group effects included ON radius (F(2,115) = 223.32, *p* < 0.001), ON length (F(2,115) = 67.02, *p* < 0.001), ON curvature (F(2,115) = 55.43, *p* < 0.001), and ON tortuosity (F(2,115) = 13.96, *p* < 0.001). In the volume metrics category, both globe volume (F(2,115) = 18.46, *p* < 0.001) and ONS volume (F(1,90) = 59.89, *p* < 0.001) showed significant group effects. (see Fig. 3)

Independent from the site (scanner/protocol) effects, several ocular metrics demonstrated significant differences between the right and left sides: Distance between globe centre and ON tip (F(1,115) = 125.75, *p* < 0.001), globe length (F(1,115) = 28.54, *p* < 0.001), globe width (F(1,115) = 46.23, *p* < 0.001), Retroorbital space width (muscle) (F(1,115) = 6.47, *p* = 0.029), Optic canal (F(1,115) = 649.39, *p* < 0.001), ONS 3 mm optimal (F(1,90) = 11.66, *p* = 0.014), ON radius (F(1,115) = 115.59, *p* < 0.001), ON length (F(1,115) = 195.10, *p* < 0.001). The detailed ANOVA results and post-hoc analysis can also be found in the Supplementary Table 1.

### Replicability of the pipeline

The *site1_T1* dataset comprised two consecutive scans acquired within a 30-minute interval, enabling further validation and verification of our analytical pipeline. Intraclass Correlation Coefficients (ICC) analysis was performed on 19 morphometric parameters, assessed bilaterally. The results demonstrated statistically significant ICCs for all morphometrics except left ON torsion. ICC values ranged from 0.41 to 0.99, indicating moderate to excellent reliability across the metrics. Detailed ICC results for individual morphometric measures are presented in Supplementary Table 2.

### Normative ocular and retroorbital metrics

Our analysis included three datasets: *site1_T1*, *site2_T1*, and *site2_T2*. The site1 dataset consisted of only males aged from 35 to 56 years (mean 43.05 y, s.d. 6.06 y). The site2 dataset comprised 17 females, 8 males, and 1 participant with missing gender information. The age had a range from 19 to 63 years (mean 32.44 y, s.d. 13.07 y) for site2 participants. The normative data and the standard deviation (s.d.) derived from these datasets are listed in Table 1. This compilation of normative values provides a comprehensive reference for the morphometrics.

**Table 1:** Normative data from the dataset with the standard deviation (s.d.).

		site1_T1 (66)	site2_T1 (26)	site2_T2 (26)	Grand mean
distance	Distance between centre of the lens and centre of the globe (mm) (R/L)	7.00 ± 0.41/6.87 ± 0.40	7.77 ± 0.67/7.79 ± 0.64	7.91 ± 0.98/7.84 ± 0.79	7.37 ± 0.07/7.28 ± 0.07
	Distance between centre of the globe and ON tip (mm) (R/L)	12.80 ± 0.34/12.87 ± 0.39	12.27 ± 0.89/12.99 ± 0.78	12.54 ± 0.93/13.18 ± 0.86	12.62 ± 0.06/12.97 ± 0.06
	Globe length (mm) (R/L)	18.99 ± 0.77/19.04 ± 0.76	18.50 ± 1.15/18.82 ± 1.19	18.20 ± 1.09/18.51 ± 1.12	18.71 ± 0.09/18.87 ± 0.09
	Distance of centre of the globe to the orbital rim (mm) (R/L)	2.24 ± 3.25/0.15 ± 4.03	1.69 ± 4.38/2.10 ± 4.11	3.74 ± 2.94/3.79 ± 2.99	2.45 ± 0.32/1.38 ± 0.38
	Distance of lens to the orbital rim (mm) (R/L)	−0.65 ± 3.49/0.20 ± 3.40	−1.23 ± 3.02/−1.36 ± 3.01	−2.10 ± 2.59/−1.81 ± 2.35	−1.10 ± 0.30/−0.59 ± 0.30
	ONSH width (mm) (R/L)	6.74 ± 0.60/7.14 ± 0.64	NaN ± NA/NaN ± NA	5.89 ± 0.39/5.53 ± 0.52	6.50 ± 0.07/6.68 ± 0.10
	Globe width (mm) (R/L)	24.45 ± 0.91/24.02 ± 0.95	23.49 ± 0.95/23.37 ± 1.05	23.48 ± 1.02/23.08 ± 1.02	24.03 ± 0.10/23.67 ± 0.10
	Retroorbital space width (muscle) (mm) (R/L)	21.92 ± 0.91/21.50 ± 1.09	20.81 ± 0.90/20.77 ± 0.75	20.93 ± 0.81/20.94 ± 0.76	21.45 ± 0.09/21.22 ± 0.09
	Retroorbital space width (bone) (mm) (R/L)	31.66 ± 0.92/31.49 ± 1.12	30.22 ± 1.38/29.80 ± 1.36	31.06 ± 1.49/31.22 ± 1.48	31.21 ± 0.12/31.06 ± 0.13
cross-sectional area	Optic canal (mm^2^) (R/L)	14.97 ± 1.84/10.38 ± 1.67	12.72 ± 2.34/7.98 ± 1.49	15.74 ± 2.57/11.76 ± 1.69	14.64 ± 0.22/10.15 ± 0.19
	ONS 3 mm (mm^2^) (R/L)	36.39 ± 4.94/38.26 ± 6.10	NaN ± NA/NaN ± NA	21.73 ± 3.79/21.16 ± 5.12	32.25 ± 0.84/33.43 ± 1.01
	ONS 3 mm optimal (mm^2^) (R/L)	34.85 ± 4.95/38.32 ± 6.50	NaN ± NA/NaN ± NA	21.52 ± 3.65/20.77 ± 4.74	31.08 ± 0.79/33.36 ± 1.04
geometry	ON radius (mm) (R/L)	1.02 ± 0.07/1.05 ± 0.08	1.47 ± 0.10/1.37 ± 0.09	1.21 ± 0.09/1.05 ± 0.12	1.16 ± 0.02/1.12 ± 0.01
	ON length (mm) (R/L)	25.24 ± 2.42/24.59 ± 2.40	20.52 ± 1.75/18.21 ± 1.51	22.24 ± 2.16/21.29 ± 2.34	23.54 ± 0.27/22.46 ± 0.32
	ON curvature (mm^−1^) (R/L)	0.11 ± 0.01/0.11 ± 0.02	0.08 ± 0.02/0.08 ± 0.01	0.09 ± 0.02/0.10 ± 0.02	0.10 ± 0.00/0.10 ± 0.00
	ON torsion (mm^−1^) (R/L)	0.10 ± 0.42/−0.05 ± 0.29	0.01 ± 0.27/0.01 ± 0.25	−0.00 ± 0.29/0.09 ± 0.36	0.06 ± 0.03/0.00 ± 0.03
	ON tortuosity (R/L)	0.04 ± 0.02/0.04 ± 0.02	0.03 ± 0.01/0.02 ± 0.01	0.03 ± 0.01/0.03 ± 0.01	0.03 ± 0.00/0.03 ± 0.00
volume	Globe (cm^3^) (R/L)	6.07 ± 0.64/5.98 ± 0.63	6.46 ± 0.79/6.15 ± 0.75	6.82 ± 0.85/7.16 ± 0.84	6.32 ± 0.07/6.27 ± 0.08
	ONS (cm^3^) (R/L)	0.50 ± 0.08/0.54 ± 0.09	NaN ± NA/NaN ± NA	0.41 ± 0.07/0.36 ± 0.07	0.47 ± 0.01/0.49 ± 0.01

This table presents the mean values and standard deviation for each cohort, along with the overall grand mean and its standard deviation (s.d.).

### The deformation map of the posterior globe segment

The deformation map of the ocular globe provides a visual representation of shape changes in the posterior globe. Our analysis of these cohorts allowed for precise quantification of physiological deviations from a perfectly spherical shape.

**Fig. 4:**
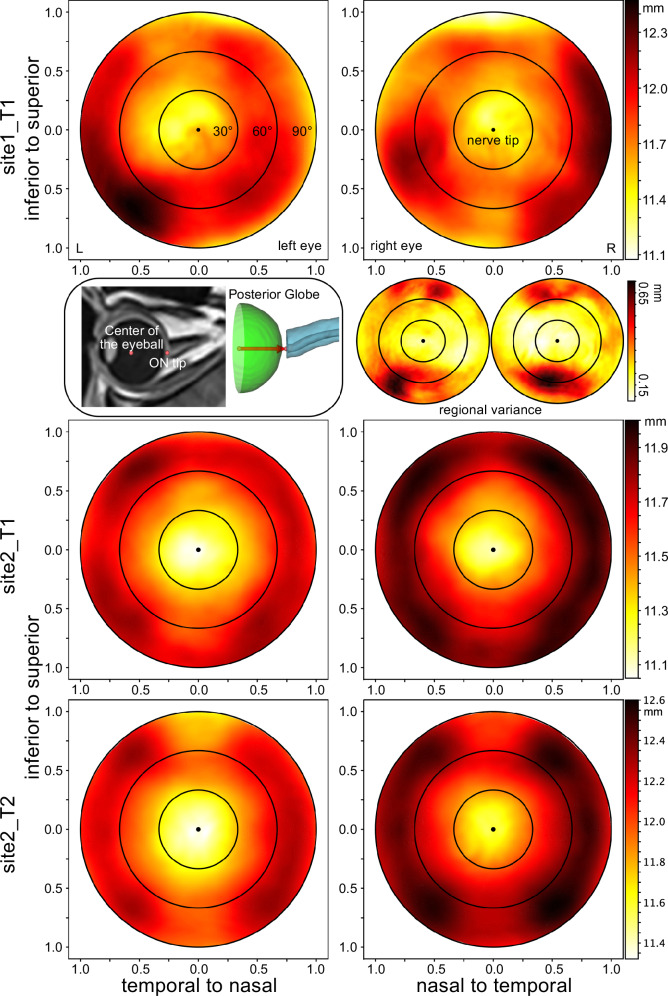
Ocular deformation maps of the posterior globe. These contour maps (0–90°) represent the radius measurements in a spherical coordination system for the entire posterior half of the eye globe. The radius is calculated from the geometric centre of the eye to the scleral wall depicted with the centre of the optic nerve tip as a reference point and defining vector for a plane perpendicular to this vector. The mean radius in the control subjects was greatest for the very temporal regions (60–90°) across the upper and lower quadrants of the maps as well as in the mid-to-lower quadrant of the nasal regions for each eye. The main sources of variance for the radius in the lower>upper third of the deformation map might reflect the insertion zones of the superior rectal and inferior oblique eye muscles in this projection.

In the *site1_T1* cohort, the central region, expanding less than the 30-degree polar angle, exhibited a relatively shorter radius. For the left eye, the greatest radius was in the temporal inferior region, whereas for the right eye, it was generally found in the for all temporal regions. The variance map from this cohort revealed that the left eye showed the largest variation in the temporal inferior area, coinciding with its greatest radius. Conversely, the right eye displayed maximum variance in the nasal inferior area (see Fig. 4). The deformation map of *site2_T1* and *site2_T2* showed similar patterns. Notably, in both cohorts, the orbit of the right eye appeared to be longer compared to that of the left eye (see Fig. 4).

## Discussion

In this study, we presented a comprehensive image processing pipeline for the segmentation and parameterisation of ocular and retroorbital structures using *MReye* imaging. The first step of our approach was the successful creation of a cohort-specific template, followed by a one-time segmentation of all regions of interest. These segmentations were then registered to individual images. Subsequently, we quantified the morphometrics of individual structures, yielding four categories of metrics: distance, cross-sectional area, geometry, and volume metrics. The pipeline and resulting metrics have potential applications in both scientific research and clinical practice, particularly in the field of ophthalmology and orbital imaging.

### Metrics selection and cohort differences

Geometry metrics quantify various parameters of the ON, including its radius, length, curvature, torsion and tortuosity. Several ophthalmic diseases can alter the geometry of the ON. For example, neurofibromatosis type 1 (NF-1) is known to manifest the ON tortuosity on the MRI images [31, 32]. Rather than merely describing the ON as tortuous, our pipeline enables the quantification of a comprehensive set of ON geometric properties. However, these metrics are not yet widely employed in clinical practice. In the scientific literature, various quantification methods have been proposed for ON geometry, such as ON deviation [8] and ON tortuosity index [33].

ONS diameter (ONSD) is a well-established indicator of elevated intracranial pressure (ICP). Traditionally, this parameter has been measured using 2D ultrasonic imaging techniques [34]. In our pipeline, we introduced a novel approach by reporting the ONS cross-sectional area instead of the conventional ONSD, leveraging the advantages of 3D segmentation in structural MRIs. While this cross-sectional estimator is not yet widely adopted in mainstream clinical practice, it has been utilised in spaceflight studies to assess ONS extension [8, 35].

In our study, we explored two distinct measurements of the ONS cross-sectional area: ONS 3 mm and ONS 3 mm optimal. The two measurements showed slight differences in our study. For site_T1, the ONS 3 mm were approximately 36.39 mm² and 38.26 mm² for the right and left eyes, respectively, while the ONS 3 mm optimal were 34.85 mm² and 38.32 mm². Despite these minor variations, both measurements yielded similar statistical results regarding group differences. Interestingly, the ONS 3 mm optimal revealed a significant difference between the right and left ONS, a finding not observed with the ONS 3 mm. This discrepancy highlights the potential sensitivity of the ONS 3 mm optimal metric in detecting subtle asymmetries in ONS morphology, suggesting it may serve as a more precise estimator for ICP changes.

Our method, based on 3D segmentation, offers greater flexibility compared to previous approaches. It allows for the calculation of cross-sectional area continuously along the ONS, providing a more comprehensive assessment of both the ON and ONS. However, it is important to note that further validation studies are needed to fully establish the clinical significance and reliability of this novel metric.

### Eye-structural laterality findings

Several morphometrics revealed significant asymmetry between the left and right ocular structures; however, these differences were accompanied by an interaction effect with imaging parameters. This interaction suggests that the observed asymmetries are partially dependent on the scanner and image contrast. For instance, we did not observe asymmetry of the distance between globe centre and ON tip in the *site1_T1* dataset, while other two datasets showed significant bilateral differences.

Among all parameters analysed in our study, only the cross-sectional area of the optic canal demonstrated consistent asymmetry across different cohorts. This finding aligns with Zhang et al.’s observations from CT images, where they reported that the minimal cross-sectional area of the right optic canal was consistently larger than the left [36]. Although our study measured only one cross-sectional plane of the optic canal, our results confirm these previously reported asymmetric patterns. However, the literature also presents some contradictory findings. Farrokhi et al. found no significant asymmetry between right and left optic canals when comparing diameters in axial, coronal, and sagittal planes from CT images [37].

The variation in findings across studies and imaging modalities emphasises the need for standardised, objective measurement protocols in ophthalmic imaging. Our pipeline, utilising 3D segmentation techniques, represents a step toward establishing such standardised protocols. This approach could enhance the comparability of future studies and advance our understanding of orbital anatomical asymmetries.

### Normative data reporting

In this study, we reported a comprehensive summary of ocular globe morphometrics, including mean and standard deviation values for each cohort and an overall grand mean. The metrics from our pipeline align closely with previous literature on ophthalmic measurements.

Bekerman et al. reported the transverse diameter of the eyeball, which corresponds to the globe width in our measurement, as 22.82 ± 1.7 mm for the right eye and 22.94 ± 1.8 mm for the left eye. Our measurements yielded slightly larger values: 24.03 ± 0.1 mm and 23.67 ± 0.1 mm for the right and left eyes, respectively. This small discrepancy may be attributed to differences in imaging contrast and study populations. Regarding the axial diameter of the eyeball, Bekerman et al. also reported 23.42 ± 1.9 mm (right eye) and 23.56 ± 1.9 mm (left eye) in emmetropic eyes. In our study, we measured the globe length as 18.71 ± 0.09 mm (right eye) and 18.87 ± 0.09 mm (left eye) [38]. It is important to note that these measurements are not directly comparable due to methodological differences. The axial diameter reported by Bekerman et al. was measured from the cornea to the sclera, while our globe length measurement was taken from the lens centre to the sclera. This difference in anatomical landmarks accounts for the shorter length observed in our study.

### Test-retest reliability

The *site1_T1* dataset provided a good opportunity to assess the test-retest reliability of our pipeline. The ICC demonstrated excellent agreement between two consecutive MRI sessions conducted 30 min apart for most metrics. However, ON torsion exhibited relatively lower agreement. Normative data revealed that torsion showed a comparatively larger standard deviation and smaller absolute values than curvature. For instance, in the *site1_T1* cohort, the mean and standard deviation for torsion in the right and left eyes were 0.10 ± 0.42 mm⁻¹ and −0.05 ± 0.29 mm⁻¹, respectively. Torsion, by definition, quantifies how a curve deviates from a planar. Given the confined space within the orbital area, one would expect ON torsion to be inherently small. The combination of low absolute values and high variability suggests that torsion may not be a reliable metric for characterising ON geometry in this context.

### Deformation map of the posterior globe

We generated displacement maps of the posterior globe to visualise mechanical load from the ONS, a potential theory for the globe flattening observed in spacefarers. Sater and his colleagues employed a slightly different computational approach for posterior globe displacement mapping, yielding results that diverged from our findings. While their analysis did not reveal ONH region shortening in preflight data, our displacement maps clearly demonstrate reduced radius in the ONH surrounding area [39]. This methodological variation and its impact on results underscores the need for standardised protocols in posterior globe displacement mapping to ensure reproducibility and facilitate direct comparisons across studies. The utility of ocular globe deformation mapping extends beyond spaceflight applications; previous research using ex vivo sheep eyes has shown significant globe deformation in response to intraocular pressure (IOP) variations between 10–20 mmHg [40].

### Limitations and outlook

Our pipeline, despite being fully automated and robust, still has some limitations that may present opportunities for further evolution.

On the topic of template specificity: Currently, we require the creation of a cohort-specific template for MRIs from different study populations. Though this approach is fully automated in our pipeline, it may not be optimal for large multisite datasets.

On the topic of available morphometric parameters: In this study, we calculated a total of 38 parameters (bilaterally) based on previous literature, complemented by novel exploratory parameters. While comprehensive, this set of metrics can be further enriched and tailored for specific research objectives.

## Conclusion

We present a robust and user-friendly pipeline for quantitative assessment of ocular and retroorbital morphometrics using structural MRIs (*MReye-Seg*). Morphometric analyses from three test cohorts revealed cohort-specific differences attributable to varying MRI contrast, as well as notable physiological asymmetries between the right and left eye. All tested metrics demonstrated very good test-retest reliability, except for torsion in the geometric evaluation. In our opinion, *MReye-Seg* is now ready for immediate implementation in both clinical practice and research settings, offering ophthalmologists enhanced diagnostic precision and monitoring capabilities across diverse patient populations. Beyond ophthalmology, this tool provides valuable applications in neurology, neurosurgery, neuroradiology, and intensive care medicine where ocular and retroorbital assessment informs clinical decision-making and advances patient care.

## Summary

### What was known before

- Ophthalmic morphometry on MRI relied predominantly on subjective manual measurements, introducing variability and limiting integration into high-volume clinical practice.
- Semi-automated tools existed only for isolated ophthalmic morphometrics but lacked the comprehensive approach needed for complete ocular and retroorbital assessment.

### What this study adds

- MReye-Seg enables automated extraction of 38 bilateral morphometric parameters with reproducibility shown by ICC > 0.8 for 79% of metrics and identifies physiological asymmetry between left and right eyes.
- This validated, open-source pipeline provides standardised quantification of ophthalmic morphometrics with minimal inter-dataset variations, establishing normative ranges for clinical interpretation across different MRI protocols.

## Supplementary information

Supplemental Material

## Data Availability

The data that support the findings of this study are available from the corresponding author, GT, upon reasonable request.
