# Supplementary material for: MReye-Seg: development and validation of an automated MRI pipeline for standardised ocular and orbital morphometrics
Source: Eye (Lond). 2025 Oct 14;39(18):3294–305. doi: 10.1038/s41433-025-04044-1 (PMC12669591; doi:10.1038/s41433-025-04044-1)
Supplement: Supplementary file 1 — Supplemental Material [file 41433_2025_4044_MOESM1_ESM.docx]

Supplementary Table 1. Post hoc results for all variables

| variable | comparison | cohort | side | cohort_pairwise | estimate | SE | df | t.ratio | P value | sig |
| --- | --- | --- | --- | --- | --- | --- | --- | --- | --- | --- |
| Distance between lens centre and globe centre (mm) | side | site1_T1 |  |  | -0,13 | 0,04 | 115 | -2,95 | 0.004 | ** |
| Distance between lens centre and globe centre (mm) | side | site2_T1 |  |  | 0,02 | 0,07 | 115 | 0,28 | 0.779 | ns |
| Distance between lens centre and globe centre (mm) | side | site2_T2 |  |  | -0,07 | 0,07 | 115 | -1 | 0.319 | ns |
| Distance between lens centre and globe centre (mm) | cohort |  | left |  | -0,92 | 0,14 | 138,18 | -6,66 | <0.001 | *** |
| Distance between lens centre and globe centre (mm) | cohort |  | left |  | -0,97 | 0,14 | 138,18 | -7,02 | <0.001 | *** |
| Distance between lens centre and globe centre (mm) | cohort |  | left |  | -0,05 | 0,17 | 138,18 | -0,3 | 0.762 | ns |
| Distance between lens centre and globe centre (mm) | cohort |  | right |  | -0,77 | 0,14 | 138,18 | -5,56 | <0.001 | *** |
| Distance between lens centre and globe centre (mm) | cohort |  | right |  | -0,91 | 0,14 | 138,18 | -6,59 | <0.001 | *** |
| Distance between lens centre and globe centre (mm) | cohort |  | right |  | -0,14 | 0,17 | 138,18 | -0,86 | 0.394 | ns |
| Distance between lens centre and globe centre (mm) | interaction |  |  | site1_T1 - site2_T1 | -0,15 | 0,08 | 115 | -1,8 | 0.221 | ns |
| Distance between lens centre and globe centre (mm) | interaction |  |  | site1_T1 - site2_T2 | -0,06 | 0,08 | 115 | -0,72 | 0.475 | ns |
| Distance between lens centre and globe centre (mm) | interaction |  |  | site2_T1 - site2_T2 | 0,09 | 0,1 | 115 | 0,91 | 0.475 | ns |
| Distance between globe centre and ON tip (mm) | side | site1_T1 |  |  | 0,07 | 0,05 | 115 | 1,39 | 0.168 | ns |
| Distance between globe centre and ON tip (mm) | side | site2_T1 |  |  | 0,72 | 0,08 | 115 | 8,85 | <0.001 | *** |
| Distance between globe centre and ON tip (mm) | side | site2_T2 |  |  | 0,65 | 0,08 | 115 | 7,91 | <0.001 | *** |
| Distance between globe centre and ON tip (mm) | cohort |  | left |  | -0,12 | 0,15 | 142,39 | -0,83 | 0.408 | ns |
| Distance between globe centre and ON tip (mm) | cohort |  | left |  | -0,31 | 0,15 | 142,39 | -2,12 | 0.108 | ns |
| Distance between globe centre and ON tip (mm) | cohort |  | left |  | -0,19 | 0,18 | 142,39 | -1,08 | 0.408 | ns |
| Distance between globe centre and ON tip (mm) | cohort |  | right |  | 0,53 | 0,15 | 142,39 | 3,61 | 0.001 | ** |
| Distance between globe centre and ON tip (mm) | cohort |  | right |  | 0,26 | 0,15 | 142,39 | 1,8 | 0.111 | ns |
| Distance between globe centre and ON tip (mm) | cohort |  | right |  | -0,27 | 0,18 | 142,39 | -1,51 | 0.134 | ns |
| Distance between globe centre and ON tip (mm) | interaction |  |  | site1_T1 - site2_T1 | -0,65 | 0,1 | 115 | -6,75 | <0.001 | *** |
| Distance between globe centre and ON tip (mm) | interaction |  |  | site1_T1 - site2_T2 | -0,57 | 0,1 | 115 | -5,96 | <0.001 | *** |
| Distance between globe centre and ON tip (mm) | interaction |  |  | site2_T1 - site2_T2 | 0,08 | 0,12 | 115 | 0,66 | 0.510 | ns |
| Globe length (mm) | side | site1_T1 |  |  | 0,05 | 0,04 | 115 | 1,19 | 0.237 | ns |
| Globe length (mm) | side | site2_T1 |  |  | 0,32 | 0,07 | 115 | 4,48 | <0.001 | *** |
| Globe length (mm) | side | site2_T2 |  |  | 0,31 | 0,07 | 115 | 4,39 | <0.001 | *** |
| Globe length (mm) | cohort |  | left |  | 0,22 | 0,22 | 123,78 | 1,01 | 0.316 | ns |
| Globe length (mm) | cohort |  | left |  | 0,53 | 0,22 | 123,78 | 2,41 | 0.053 | ns |
| Globe length (mm) | cohort |  | left |  | 0,31 | 0,26 | 123,78 | 1,17 | 0.316 | ns |
| Globe length (mm) | cohort |  | right |  | 0,48 | 0,22 | 123,78 | 2,22 | 0.042 | * |
| Globe length (mm) | cohort |  | right |  | 0,78 | 0,22 | 123,78 | 3,59 | 0.001 | ** |
| Globe length (mm) | cohort |  | right |  | 0,3 | 0,26 | 123,78 | 1,15 | 0.254 | ns |
| Globe length (mm) | interaction |  |  | site1_T1 - site2_T1 | -0,26 | 0,08 | 115 | -3,16 | 0.004 | ** |
| Globe length (mm) | interaction |  |  | site1_T1 - site2_T2 | -0,26 | 0,08 | 115 | -3,09 | 0.004 | ** |
| Globe length (mm) | interaction |  |  | site2_T1 - site2_T2 | 0,01 | 0,1 | 115 | 0,06 | 0.951 | ns |
| Distance of globe centre to the orbital rim (mm) | side | site1_T1 |  |  | -2,09 | 0,42 | 115 | -4,97 | <0.001 | *** |
| Distance of globe centre to the orbital rim (mm) | side | site2_T1 |  |  | 0,41 | 0,67 | 115 | 0,61 | 0.543 | ns |
| Distance of globe centre to the orbital rim (mm) | side | site2_T2 |  |  | 0,05 | 0,67 | 115 | 0,07 | 0.944 | ns |
| Distance of globe centre to the orbital rim (mm) | cohort |  | left |  | -1,95 | 0,85 | 174,19 | -2,3 | 0.034 | * |
| Distance of globe centre to the orbital rim (mm) | cohort |  | left |  | -3,64 | 0,85 | 174,19 | -4,29 | <0.001 | *** |
| Distance of globe centre to the orbital rim (mm) | cohort |  | left |  | -1,69 | 1,02 | 174,19 | -1,66 | 0.098 | ns |
| Distance of globe centre to the orbital rim (mm) | cohort |  | right |  | 0,55 | 0,85 | 174,19 | 0,64 | 0.520 | ns |
| Distance of globe centre to the orbital rim (mm) | cohort |  | right |  | -1,51 | 0,85 | 174,19 | -1,78 | 0.116 | ns |
| Distance of globe centre to the orbital rim (mm) | cohort |  | right |  | -2,05 | 1,02 | 174,19 | -2,02 | 0.116 | ns |
| Distance of globe centre to the orbital rim (mm) | interaction |  |  | site1_T1 - site2_T1 | -2,5 | 0,79 | 115 | -3,16 | 0.006 | ** |
| Distance of globe centre to the orbital rim (mm) | interaction |  |  | site1_T1 - site2_T2 | -2,13 | 0,79 | 115 | -2,7 | 0.012 | * |
| Distance of globe centre to the orbital rim (mm) | interaction |  |  | site2_T1 - site2_T2 | 0,36 | 0,95 | 115 | 0,38 | 0.703 | ns |
| Distance of lens centre to the orbital rim (mm) | side | site1_T1 |  |  | 0,85 | 0,35 | 115 | 2,44 | 0.016 | * |
| Distance of lens centre to the orbital rim (mm) | side | site2_T1 |  |  | -0,13 | 0,55 | 115 | -0,24 | 0.810 | ns |
| Distance of lens centre to the orbital rim (mm) | side | site2_T2 |  |  | 0,29 | 0,55 | 115 | 0,53 | 0.597 | ns |
| Distance of lens centre to the orbital rim (mm) | cohort |  | left |  | 1,56 | 0,73 | 168,65 | 2,13 | 0.052 | ns |
| Distance of lens centre to the orbital rim (mm) | cohort |  | left |  | 2,01 | 0,73 | 168,65 | 2,75 | 0.020 | * |
| Distance of lens centre to the orbital rim (mm) | cohort |  | left |  | 0,45 | 0,88 | 168,65 | 0,51 | 0.609 | ns |
| Distance of lens centre to the orbital rim (mm) | cohort |  | right |  | 0,58 | 0,73 | 168,65 | 0,79 | 0.430 | ns |
| Distance of lens centre to the orbital rim (mm) | cohort |  | right |  | 1,46 | 0,73 | 168,65 | 1,99 | 0.145 | ns |
| Distance of lens centre to the orbital rim (mm) | cohort |  | right |  | 0,88 | 0,88 | 168,65 | 1 | 0.430 | ns |
| Distance of lens centre to the orbital rim (mm) | interaction |  |  | site1_T1 - site2_T1 | 0,98 | 0,65 | 115 | 1,5 | 0.406 | ns |
| Distance of lens centre to the orbital rim (mm) | interaction |  |  | site1_T1 - site2_T2 | 0,56 | 0,65 | 115 | 0,85 | 0.587 | ns |
| Distance of lens centre to the orbital rim (mm) | interaction |  |  | site2_T1 - site2_T2 | -0,43 | 0,78 | 115 | -0,55 | 0.587 | ns |
| ONSH width (mm) | side | site1_T1 |  |  | 0,39 | 0,05 | 90 | 7,26 | <0.001 | *** |
| ONSH width (mm) | side | site2_T2 |  |  | -0,36 | 0,09 | 90 | -4,21 | <0.001 | *** |
| ONSH width (mm) | cohort |  | left |  | 1,61 | 0,13 | 118,93 | 11,92 | <0.001 | *** |
| ONSH width (mm) | cohort |  | right |  | 0,85 | 0,13 | 118,93 | 6,32 | <0.001 | *** |
| ONSH width (mm) | interaction |  |  | site1_T1 - site2_T2 | 0,75 | 0,1 | 90 | 7,43 | <0.001 | *** |
| Globe width (mm) | side | site1_T1 |  |  | -0,44 | 0,06 | 115 | -7,51 | <0.001 | *** |
| Globe width (mm) | side | site2_T1 |  |  | -0,12 | 0,09 | 115 | -1,33 | 0.186 | ns |
| Globe width (mm) | side | site2_T2 |  |  | -0,39 | 0,09 | 115 | -4,27 | <0.001 | *** |
| Globe width (mm) | cohort |  | left |  | 0,65 | 0,22 | 129,54 | 2,9 | 0.007 | ** |
| Globe width (mm) | cohort |  | left |  | 0,94 | 0,22 | 129,54 | 4,19 | <0.001 | *** |
| Globe width (mm) | cohort |  | left |  | 0,29 | 0,27 | 129,54 | 1,08 | 0.283 | ns |
| Globe width (mm) | cohort |  | right |  | 0,96 | 0,22 | 129,54 | 4,3 | <0.001 | *** |
| Globe width (mm) | cohort |  | right |  | 0,98 | 0,22 | 129,54 | 4,37 | <0.001 | *** |
| Globe width (mm) | cohort |  | right |  | 0,02 | 0,27 | 129,54 | 0,06 | 0.950 | ns |
| Globe width (mm) | interaction |  |  | site1_T1 - site2_T1 | -0,31 | 0,11 | 115 | -2,86 | 0.015 | * |
| Globe width (mm) | interaction |  |  | site1_T1 - site2_T2 | -0,04 | 0,11 | 115 | -0,38 | 0.708 | ns |
| Globe width (mm) | interaction |  |  | site2_T1 - site2_T2 | 0,27 | 0,13 | 115 | 2,08 | 0.060 | ns |
| Retroorbital space width (muscle) (mm) | side | site1_T1 |  |  | -0,41 | 0,07 | 115 | -6,02 | <0.001 | *** |
| Retroorbital space width (muscle) (mm) | side | site2_T1 |  |  | -0,03 | 0,11 | 115 | -0,29 | 0.773 | ns |
| Retroorbital space width (muscle) (mm) | side | site2_T2 |  |  | 0,01 | 0,11 | 115 | 0,13 | 0.897 | ns |
| Retroorbital space width (muscle) (mm) | cohort |  | left |  | 0,73 | 0,21 | 137,66 | 3,41 | 0.003 | ** |
| Retroorbital space width (muscle) (mm) | cohort |  | left |  | 0,56 | 0,21 | 137,66 | 2,61 | 0.015 | * |
| Retroorbital space width (muscle) (mm) | cohort |  | left |  | -0,17 | 0,26 | 137,66 | -0,67 | 0.506 | ns |
| Retroorbital space width (muscle) (mm) | cohort |  | right |  | 1,11 | 0,21 | 137,66 | 5,19 | <0.001 | *** |
| Retroorbital space width (muscle) (mm) | cohort |  | right |  | 0,99 | 0,21 | 137,66 | 4,6 | <0.001 | *** |
| Retroorbital space width (muscle) (mm) | cohort |  | right |  | -0,13 | 0,26 | 137,66 | -0,49 | 0.626 | ns |
| Retroorbital space width (muscle) (mm) | interaction |  |  | site1_T1 - site2_T1 | -0,38 | 0,13 | 115 | -2,96 | 0.006 | ** |
| Retroorbital space width (muscle) (mm) | interaction |  |  | site1_T1 - site2_T2 | -0,43 | 0,13 | 115 | -3,31 | 0.004 | ** |
| Retroorbital space width (muscle) (mm) | interaction |  |  | site2_T1 - site2_T2 | -0,05 | 0,15 | 115 | -0,3 | 0.768 | ns |
| Retroorbital space width (bone) (mm) | side | site1_T1 |  |  | -0,17 | 0,08 | 115 | -2,06 | 0.041 | * |
| Retroorbital space width (bone) (mm) | side | site2_T1 |  |  | -0,42 | 0,13 | 115 | -3,3 | 0.001 | ** |
| Retroorbital space width (bone) (mm) | side | site2_T2 |  |  | 0,15 | 0,13 | 115 | 1,21 | 0.228 | ns |
| Retroorbital space width (bone) (mm) | cohort |  | left |  | 1,69 | 0,28 | 132,51 | 5,99 | <0.001 | *** |
| Retroorbital space width (bone) (mm) | cohort |  | left |  | 0,28 | 0,28 | 132,51 | 0,98 | 0.329 | ns |
| Retroorbital space width (bone) (mm) | cohort |  | left |  | -1,41 | 0,34 | 132,51 | -4,18 | <0.001 | *** |
| Retroorbital space width (bone) (mm) | cohort |  | right |  | 1,43 | 0,28 | 132,51 | 5,09 | <0.001 | *** |
| Retroorbital space width (bone) (mm) | cohort |  | right |  | 0,6 | 0,28 | 132,51 | 2,11 | 0.037 | * |
| Retroorbital space width (bone) (mm) | cohort |  | right |  | -0,84 | 0,34 | 132,51 | -2,48 | 0.021 | * |
| Retroorbital space width (bone) (mm) | interaction |  |  | site1_T1 - site2_T1 | 0,26 | 0,15 | 115 | 1,7 | 0.093 | ns |
| Retroorbital space width (bone) (mm) | interaction |  |  | site1_T1 - site2_T2 | -0,32 | 0,15 | 115 | -2,12 | 0.054 | ns |
| Retroorbital space width (bone) (mm) | interaction |  |  | site2_T1 - site2_T2 | -0,57 | 0,18 | 115 | -3,19 | 0.006 | ** |
| Optic canal (mm^2^) | side | site1_T1 |  |  | -4,59 | 0,21 | 115 | -21,93 | <0.001 | *** |
| Optic canal (mm^2^) | side | site2_T1 |  |  | -4,74 | 0,33 | 115 | -14,22 | <0.001 | *** |
| Optic canal (mm^2^) | side | site2_T2 |  |  | -3,98 | 0,33 | 115 | -11,93 | <0.001 | *** |
| Optic canal (mm^2^) | cohort |  | left |  | 2,4 | 0,44 | 169,14 | 5,46 | <0.001 | *** |
| Optic canal (mm^2^) | cohort |  | left |  | -1,38 | 0,44 | 169,14 | -3,14 | 0.002 | ** |
| Optic canal (mm^2^) | cohort |  | left |  | -3,78 | 0,53 | 169,14 | -7,18 | <0.001 | *** |
| Optic canal (mm^2^) | cohort |  | right |  | 2,25 | 0,44 | 169,14 | 5,11 | <0.001 | *** |
| Optic canal (mm^2^) | cohort |  | right |  | -0,77 | 0,44 | 169,14 | -1,76 | 0.081 | ns |
| Optic canal (mm^2^) | cohort |  | right |  | -3,02 | 0,53 | 169,14 | -5,74 | <0.001 | *** |
| Optic canal (mm^2^) | interaction |  |  | site1_T1 - site2_T1 | 0,15 | 0,39 | 115 | 0,38 | 0.704 | ns |
| Optic canal (mm^2^) | interaction |  |  | site1_T1 - site2_T2 | -0,61 | 0,39 | 115 | -1,55 | 0.185 | ns |
| Optic canal (mm^2^) | interaction |  |  | site2_T1 - site2_T2 | -0,76 | 0,47 | 115 | -1,61 | 0.185 | ns |
| ONS 3mm (mm^2^) | side | site1_T1 |  |  | 1,87 | 0,49 | 90 | 3,82 | <0.001 | *** |
| ONS 3mm (mm^2^) | side | site2_T2 |  |  | -0,57 | 0,78 | 90 | -0,73 | 0.465 | ns |
| ONS 3mm (mm^2^) | cohort |  | left |  | 17,1 | 1,22 | 118,97 | 13,98 | <0.001 | *** |
| ONS 3mm (mm^2^) | cohort |  | right |  | 14,66 | 1,22 | 118,97 | 11,99 | <0.001 | *** |
| ONS 3mm (mm^2^) | interaction |  |  | site1_T1 - site2_T2 | 2,44 | 0,92 | 90 | 2,65 | 0.010 | ** |
| ONS 3mm optimal (mm^2^) | side | site1_T1 |  |  | 3,47 | 0,5 | 90 | 6,89 | <0.001 | *** |
| ONS 3mm optimal (mm^2^) | side | site2_T2 |  |  | -0,75 | 0,8 | 90 | -0,94 | 0.350 | ns |
| ONS 3mm optimal (mm^2^) | cohort |  | left |  | 17,55 | 1,25 | 119,41 | 14,06 | <0.001 | *** |
| ONS 3mm optimal (mm^2^) | cohort |  | right |  | 13,33 | 1,25 | 119,41 | 10,68 | <0.001 | *** |
| ONS 3mm optimal (mm^2^) | interaction |  |  | site1_T1 - site2_T2 | 4,22 | 0,95 | 90 | 4,46 | <0.001 | *** |
| ON radius (mm) | side | site1_T1 |  |  | 0,03 | 0,01 | 115 | 4,27 | <0.001 | *** |
| ON radius (mm) | side | site2_T1 |  |  | -0,1 | 0,01 | 115 | -7,92 | <0.001 | *** |
| ON radius (mm) | side | site2_T2 |  |  | -0,16 | 0,01 | 115 | -12,46 | <0.001 | *** |
| ON radius (mm) | cohort |  | left |  | -0,32 | 0,02 | 152,37 | -15,95 | <0.001 | *** |
| ON radius (mm) | cohort |  | left |  | 0 | 0,02 | 152,37 | -0,09 | 0.930 | ns |
| ON radius (mm) | cohort |  | left |  | 0,32 | 0,02 | 152,37 | 13,24 | <0.001 | *** |
| ON radius (mm) | cohort |  | right |  | -0,45 | 0,02 | 152,37 | -22,75 | <0.001 | *** |
| ON radius (mm) | cohort |  | right |  | -0,19 | 0,02 | 152,37 | -9,79 | <0.001 | *** |
| ON radius (mm) | cohort |  | right |  | 0,26 | 0,02 | 152,37 | 10,82 | <0.001 | *** |
| ON radius (mm) | interaction |  |  | site1_T1 - site2_T1 | 0,14 | 0,02 | 115 | 8,98 | <0.001 | *** |
| ON radius (mm) | interaction |  |  | site1_T1 - site2_T2 | 0,19 | 0,02 | 115 | 12,82 | <0.001 | *** |
| ON radius (mm) | interaction |  |  | site2_T1 - site2_T2 | 0,06 | 0,02 | 115 | 3,21 | 0.002 | ** |
| ON length (mm) | side | site1_T1 |  |  | -0,65 | 0,11 | 115 | -5,77 | <0.001 | *** |
| ON length (mm) | side | site2_T1 |  |  | -2,31 | 0,18 | 115 | -12,89 | <0.001 | *** |
| ON length (mm) | side | site2_T2 |  |  | -0,95 | 0,18 | 115 | -5,27 | <0.001 | *** |
| ON length (mm) | cohort |  | left |  | 6,38 | 0,52 | 125,11 | 12,36 | <0.001 | *** |
| ON length (mm) | cohort |  | left |  | 3,29 | 0,52 | 125,11 | 6,38 | <0.001 | *** |
| ON length (mm) | cohort |  | left |  | -3,09 | 0,62 | 125,11 | -4,99 | <0.001 | *** |
| ON length (mm) | cohort |  | right |  | 4,71 | 0,52 | 125,11 | 9,13 | <0.001 | *** |
| ON length (mm) | cohort |  | right |  | 3 | 0,52 | 125,11 | 5,81 | <0.001 | *** |
| ON length (mm) | cohort |  | right |  | -1,72 | 0,62 | 125,11 | -2,78 | 0.006 | ** |
| ON length (mm) | interaction |  |  | site1_T1 - site2_T1 | 1,66 | 0,21 | 115 | 7,85 | <0.001 | *** |
| ON length (mm) | interaction |  |  | site1_T1 - site2_T2 | 0,3 | 0,21 | 115 | 1,4 | 0.164 | ns |
| ON length (mm) | interaction |  |  | site2_T1 - site2_T2 | -1,37 | 0,25 | 115 | -5,39 | <0.001 | *** |
| ON curvature (mm^-1^) | side | site1_T1 |  |  | 0 | 0 | 115 | 1 | 0.321 | ns |
| ON curvature (mm^-1^) | side | site2_T1 |  |  | -0,01 | 0 | 115 | -2,03 | 0.044 | * |
| ON curvature (mm^-1^) | side | site2_T2 |  |  | 0,01 | 0 | 115 | 3,71 | <0.001 | *** |
| ON curvature (mm^-1^) | cohort |  | left |  | 0,04 | 0 | 202,53 | 9,88 | <0.001 | *** |
| ON curvature (mm^-1^) | cohort |  | left |  | 0,01 | 0 | 202,53 | 2,8 | 0.006 | ** |
| ON curvature (mm^-1^) | cohort |  | left |  | -0,03 | 0 | 202,53 | -5,91 | <0.001 | *** |
| ON curvature (mm^-1^) | cohort |  | right |  | 0,03 | 0 | 202,53 | 7,35 | <0.001 | *** |
| ON curvature (mm^-1^) | cohort |  | right |  | 0,02 | 0 | 202,53 | 5,74 | <0.001 | *** |
| ON curvature (mm^-1^) | cohort |  | right |  | -0,01 | 0 | 202,53 | -1,34 | 0.181 | ns |
| ON curvature (mm^-1^) | interaction |  |  | site1_T1 - site2_T1 | 0,01 | 0 | 115 | 2,25 | 0.026 | * |
| ON curvature (mm^-1^) | interaction |  |  | site1_T1 - site2_T2 | -0,01 | 0 | 115 | -2,61 | 0.015 | * |
| ON curvature (mm^-1^) | interaction |  |  | site2_T1 - site2_T2 | -0,02 | 0,01 | 115 | -4,06 | <0.001 | *** |
| ON tortuosity | side | site1_T1 |  |  | 0 | 0 | 115 | 1,14 | 0.255 | ns |
| ON tortuosity | side | site2_T1 |  |  | -0,01 | 0 | 115 | -2,69 | 0.008 | ** |
| ON tortuosity | side | site2_T2 |  |  | 0 | 0 | 115 | 0,32 | 0.753 | ns |
| ON tortuosity | cohort |  | left |  | 0,02 | 0 | 157,91 | 5,59 | <0.001 | *** |
| ON tortuosity | cohort |  | left |  | 0,01 | 0 | 157,91 | 3,43 | 0.001 | ** |
| ON tortuosity | cohort |  | left |  | -0,01 | 0 | 157,91 | -1,8 | 0.073 | ns |
| ON tortuosity | cohort |  | right |  | 0,01 | 0 | 157,91 | 3,26 | 0.003 | ** |
| ON tortuosity | cohort |  | right |  | 0,01 | 0 | 157,91 | 3,16 | 0.003 | ** |
| ON tortuosity | cohort |  | right |  | 0 | 0 | 157,91 | -0,09 | 0.929 | ns |
| ON tortuosity | interaction |  |  | site1_T1 - site2_T1 | 0,01 | 0 | 115 | 2,89 | 0.014 | * |
| ON tortuosity | interaction |  |  | site1_T1 - site2_T2 | 0 | 0 | 115 | 0,34 | 0.734 | ns |
| ON tortuosity | interaction |  |  | site2_T1 - site2_T2 | -0,01 | 0 | 115 | -2,13 | 0.053 | ns |
| Globe (cm^3^) | side | site1_T1 |  |  | -0,1 | 0,02 | 115 | -5,74 | <0.001 | *** |
| Globe (cm^3^) | side | site2_T1 |  |  | -0,31 | 0,03 | 115 | -11,48 | <0.001 | *** |
| Globe (cm^3^) | side | site2_T2 |  |  | 0,33 | 0,03 | 115 | 12,41 | <0.001 | *** |
| Globe (cm^3^) | cohort |  | left |  | -0,17 | 0,17 | 117,14 | -1,06 | 0.293 | ns |
| Globe (cm^3^) | cohort |  | left |  | -1,18 | 0,17 | 117,14 | -7,15 | <0.001 | *** |
| Globe (cm^3^) | cohort |  | left |  | -1,01 | 0,2 | 117,14 | -5,08 | <0.001 | *** |
| Globe (cm^3^) | cohort |  | right |  | -0,39 | 0,17 | 117,14 | -2,34 | 0.032 | * |
| Globe (cm^3^) | cohort |  | right |  | -0,75 | 0,17 | 117,14 | -4,54 | <0.001 | *** |
| Globe (cm^3^) | cohort |  | right |  | -0,36 | 0,2 | 117,14 | -1,84 | 0.068 | ns |
| Globe (cm^3^) | interaction |  |  | site1_T1 - site2_T1 | 0,21 | 0,03 | 115 | 6,68 | <0.001 | *** |
| Globe (cm^3^) | interaction |  |  | site1_T1 - site2_T2 | -0,43 | 0,03 | 115 | -13,56 | <0.001 | *** |
| Globe (cm^3^) | interaction |  |  | site2_T1 - site2_T2 | -0,64 | 0,04 | 115 | -16,89 | <0.001 | *** |
| ONS (cm^3^) | side | site1_T1 |  |  | 0,04 | 0 | 90 | 8,29 | <0.001 | *** |
| ONS (cm^3^) | side | site2_T2 |  |  | -0,04 | 0,01 | 90 | -5,94 | <0.001 | *** |
| ONS (cm^3^) | cohort |  | left |  | 0,18 | 0,02 | 99,24 | 9,3 | <0.001 | *** |
| ONS (cm^3^) | cohort |  | right |  | 0,1 | 0,02 | 99,24 | 5,12 | <0.001 | *** |
| ONS (cm^3^) | interaction |  |  | site1_T1 - site2_T2 | 0,08 | 0,01 | 90 | 9,44 | <0.001 | *** |

Supplementary Table 2. ICC of all the measured parameters from site1_T1 cohort in a consecutive session with half an hour apart. This table presents the intraclass correlation coefficient (ICC) values derived from two consecutive imaging sessions of the site1_T1 cohort. The MRI scans were acquired 30 minutes apart on the same participant. Analysis of this data revealed excellent agreement between the two consecutive images for most metrics. However, the left-side optic nerve (ON) torsion measurements did not show statistically significant reproducibility.

|  | | Intraclass correlation (ICC) | 95% Confidence Interval (CI) | | F test | | | |
| --- | --- | --- | --- | --- | --- | --- | --- | --- |
|  |  |  | Lower bound | Upper bound | Fvalue | *df1* | *df2* | Sig |
| Distance | Distance between lens centre and globe centre (mm) (R) | 0.95 | 0.89 | 0.98 | 37.21 | 27 | 27.33 | <0.001 *** |
|  | Distance between lens centre and globe centre (mm) (L) | 0.95 | 0.89 | 0.98 | 36.97 | 27 | 27.05 | <0.001 *** |
|  | Distance between globe centre and ON tip (mm) (R) | 0.75 | 0.5 | 0.88 | 7.91 | 27 | 20.26 | <0.001 *** |
|  | Distance between globe centre and ON tip (mm) (L) | 0.77 | 0.57 | 0.89 | 7.63 | 27 | 27 | <0.001 *** |
|  | Globe length (mm) (R) | 0.97 | 0.94 | 0.99 | 67.51 | 27 | 27 | <0.001 *** |
|  | Globe length (mm) (L) | 0.94 | 0.88 | 0.97 | 31.67 | 27 | 27.16 | <0.001 *** |
|  | Distance of globe centre to the orbital rim (mm) (R) | 0.53 | 0.22 | 0.75 | 3.47 | 27 | 26.74 | <0.001 *** |
|  | Distance of globe centre to the orbital rim (mm) (L) | 0.57 | 0.25 | 0.77 | 3.54 | 27 | 27.27 | <0.001 *** |
|  | Distance of lens centre to the orbital rim (mm) (R) | 0.84 | 0.69 | 0.92 | 11.73 | 27 | 28 | <0.001 *** |
|  | Distance of lens centre to the orbital rim (mm) (L) | 0.77 | 0.55 | 0.89 | 7.34 | 27 | 27.01 | <0.001 *** |
|  | ONSH width (mm) (R) | 0.8 | 0.6 | 0.9 | 10.04 | 27 | 22.63 | <0.001 *** |
|  | ONSH width (mm) (L) | 0.82 | 0.62 | 0.91 | 10.96 | 27 | 21.93 | <0.001 *** |
|  | Globe width (mm) (R) | 0.98 | 0.96 | 0.99 | 102.85 | 27 | 25.89 | <0.001 *** |
|  | Globe width (mm) (L) | 0.98 | 0.95 | 0.99 | 123.4 | 27 | 15.5 | <0.001 *** |
|  | Retroorbital space width (muscle) (mm) (R) | 0.97 | 0.93 | 0.99 | 60.01 | 27 | 27.56 | <0.001 *** |
|  | Retroorbital space width (muscle) (mm) (L) | 0.96 | 0.92 | 0.98 | 49.81 | 27 | 27.1 | <0.001 *** |
|  | Retroorbital space width (bone) (mm) (R) | 0.98 | 0.96 | 0.99 | 105.35 | 27 | 27.41 | <0.001 *** |
|  | Retroorbital space width (bone) (mm) (L) | 0.98 | 0.97 | 0.99 | 123.69 | 27 | 27.05 | <0.001 *** |
| cross-sectional area | Optic canal (mm^2^) (R) | 0.95 | 0.89 | 0.98 | 35.59 | 27 | 27.22 | <0.001 *** |
|  | Optic canal (mm^2^) (L) | 0.92 | 0.84 | 0.96 | 23.28 | 27 | 27.04 | <0.001 *** |
|  | ONS 3mm (mm^2^) (R) | 0.67 | 0.02 | 0.88 | 10.06 | 27 | 3.81 | 0.021 * |
|  | ONS 3mm (mm^2^) (L) | 0.63 | 0.09 | 0.85 | 7.26 | 27 | 5.76 | 0.012 * |
|  | ONS 3mm optimal (mm^2^) (R) | 0.71 | 0.06 | 0.9 | 11.58 | 27 | 3.82 | 0.016 * |
|  | ONS 3mm optimal (mm^2^) (L) | 0.63 | 0.11 | 0.84 | 6.89 | 27 | 6.36 | 0.009 ** |
| Geometry | ON radius (mm) (R) | 0.82 | 0.55 | 0.92 | 13.65 | 27 | 10.92 | <0.001 *** |
|  | ON radius (mm) (L) | 0.8 | 0.49 | 0.92 | 12.6 | 27 | 9.64 | <0.001 *** |
|  | ON length (mm) (R) | 0.98 | 0.92 | 0.99 | 111.22 | 27 | 9.42 | <0.001 *** |
|  | ON length (mm) (L) | 0.97 | 0.95 | 0.99 | 78.32 | 27 | 27.99 | <0.001 *** |
|  | ON curvature (mm^-1^) (R) | 0.52 | 0.2 | 0.74 | 3.28 | 27 | 27.11 | 0.001 ** |
|  | ON curvature (mm^-1^) (L) | 0.67 | 0.4 | 0.83 | 4.95 | 27 | 27.43 | <0.001 *** |
|  | ON torsion (mm^-1^) (R) | 0.41 | 0.04 | 0.68 | 2.34 | 27 | 27.03 | 0.015 * |
|  | ON torsion (mm^-1^) (L) | 0.27 | -0.1 | 0.58 | 1.76 | 27 | 27.65 | 0.073 ns |
|  | ON tortuosity (R) | 0.97 | 0.94 | 0.99 | 62.21 | 27 | 27.02 | <0.001 *** |
|  | ON tortuosity (L) | 0.92 | 0.84 | 0.96 | 23.29 | 27 | 27.07 | <0.001 *** |
| Volume | Globe (cm^3^) (R) | 0.99 | 0.97 | 0.99 | 138.3 | 27 | 26.89 | <0.001 *** |
|  | Globe (cm^3^) (L) | 0.98 | 0.96 | 0.99 | 125.17 | 27 | 21.92 | <0.001 *** |
|  | ONS (cm^3^) (R) | 0.84 | 0.23 | 0.95 | 24.2 | 27 | 3.48 | 0.006 ** |
|  | ONS (cm^3^) (L) | 0.8 | 0.27 | 0.93 | 15.75 | 27 | 4.74 | 0.004 ** |
